# Upper Limb End-Effector Force Estimation During Multi-Muscle Isometric Contraction Tasks Using HD-sEMG and Deep Belief Network

**DOI:** 10.3389/fnins.2020.00450

**Published:** 2020-05-07

**Authors:** Ruochen Hu, Xiang Chen, Shuai Cao, Xu Zhang, Xun Chen

**Affiliations:** Department of Electronic Science and Technology, University of Science and Technology of China, Hefei, China

**Keywords:** multi-muscle isometric contraction, end-effector force estimation, high-density surface electromyography, deep belief network, mean impact value

## Abstract

In this study, research was carried out on the end-effector force estimation of two representative multi-muscle contraction tasks: elbow flexion and palm-pressing. The aim was to ascertain whether an individual muscle or a combination of muscles is more suitable for the end-effector force estimation. High-density surface electromyography (HD-sEMG) signals were collected from four primary muscle areas of the upper arm and forearm: the biceps brachii (BB), brachialis (BR), triceps brachii (TB), brachioradialis (BRD), and extensor digitorum communis (EDC). The wrist pulling and palm-pressing forces were measured in elbow flexion and palm-pressing tasks, respectively. The deep belief network (DBN) was adopted to establish the relation between HD-sEMG and the measured force. The representative signals of the four primary areas, which were considered as the input signal of the force estimation model, were extracted by HD-sEMG using the principle component analysis (PCA) algorithm, and fed separately or together into the DBN. An index termed mean impact value (MIV) was proposed to describe the priority of different muscle groups for estimating the end-effector force. The experimental results demonstrated that, in multi-muscle isometric contraction tasks, the dominant muscles with the highest activation degree could track variations in the end-effector force more effectively, and are more suitable than a combination of muscles. The main contributions of this research are as follows: (1) To fuse the activation information from different muscles effectively, DBN was adopted to establish the relationship between HD-sEMG and the generated force, and achieved highly accurate force estimation. (2) Based on the well-trained DBN force estimation model, an index termed MIV was presented to evaluate the priority of muscles for estimating the generated force.

## Introduction

In general, it is challenging to measure the muscle force produced by skeletal muscle contraction accurately. The direct measurement method is commonly used for accurate determination of muscle force, wherein mechanical sensors are surgically placed in the tendons of skeletal muscles (Dennerlein et al., 1997; Finni et al., 1998; Huang and Gu, 2008). For example, Huang and Gu (2008) transplanted photoconductive devices into human muscle to measure muscle force directly. Finni et al. (1998) placed an optic fiber inside a volunteer’s tendon to collect the Achilles tendon force of gait. The disadvantage of direct measurement is that it is invasive, thereby limiting its scope of application. Therefore, indirect measurement methods, which are generally non-invasive, have been extensively employed to estimate the muscle force in related applications (Ayusawa et al., 2014; Martin et al., 2018). For example, in the work of Martin et al. (2018), a strain pressure sensor, which is implantable, extensible, and biodegradable, was presented to monitor patients’ mechanical force on tendons after a surgical repair period. Ayusawa et al. (2014) used a high-speed and high-precision video motion capture device to obtain human joint kinematic parameters. They then established a mechanical equilibrium equation between the joint and muscle forces based on inverse kinematics (Ayusawa et al., 2014).

Surface electromyography (sEMG), which is a non-invasive measurement method, has garnered particular interest for its advantages of safety, low cost, and convenient operation. In the research fields of biomechanics and kinesiology (Zajac et al., 2002; Christophy et al., 2012), physical rehabilitation, and myoelectric prostheses (Zheng et al., 1998; Heo et al., 2012), sEMG has been widely used to estimate muscle activation level and contraction force (Disselhorst-Klug et al., 2009; Li et al., 2014; Naik and Nguyen, 2015). The end-effector force estimation based on sEMG mainly includes two key procedures: muscle activation information extraction from raw sEMG signals, and establishment of a force estimation model. In early studies, the muscle activation information that was used to estimate the end-effector force was extracted from an individual channel or several channels of sEMG signals (Hayashibe and Guiraud, 2013; Cao et al., 2015). For example, Mobasser et al. (2007) placed two sEMG electrodes on the biceps brachii (BB) and triceps brachii (TB) to perform force estimation at the wrist during the elbow flexion–extension task. However, owing to the heterogeneity in the spatial distribution of muscle activation, the sEMG signals detected by the discrete electrodes could not effectively reflect the contraction characteristics of the whole muscle, thereby limiting the force estimation accuracy. In recent years, high-density surface electromyography (HD-sEMG), which is capable of collecting substantial amount of spatial muscle activation information, has demonstrated remarkable performance in related applications, particularly in improving force estimation precision (Staudenmann et al., 2005, 2006; Rojas-Martinez et al., 2012). In a series of related studies, Staudenmann et al. validated the good performance of HD-sEMG in the end-effector force estimation (Staudenmann et al., 2005, 2006, 2009). Huang et al. (2017) also realized highly accurate estimation of contraction force of the biceps brachii during elbow flexion task, by extracting the muscle activation information from HD-sEMG signals.

In most researches on sEMG-based force estimation, simple contraction tasks and individual skeletal muscles were involved. Taking the elbow flexion/extension task as an example, the BB was regarded as the main driving muscle in some studies (Huang et al., 2017; Xu et al., 2018), however, TB was regarded as the main driving muscle in some other researches (Staudenmann et al., 2005, 2006). It has been established that both complex and simple human motion tasks generally involve the contraction of multiple pieces of skeletal muscles, and the phenomenon of muscle co-contraction or muscle synergy patterns appears occasionally (Amarantini et al., 2010; Atoufi et al., 2013). Force estimation based on an individual muscle is conveniently realized, and is established as being practical for certain applications. However, there may be a few theoretical limitations when considering only an individual muscle. To improve the force estimation accuracy, a few researchers have attempted to explore force estimation frameworks based on multiple muscles (Luh et al., 1999; Hoozemans and van Dieen, 2005; Mobasser et al., 2007; Bai and Chew, 2013; Al Harrach et al., 2017; Chen X. et al., 2018). In the work of Harrach et al. (Al Harrach et al., 2017), three elbow flexor muscles, namely, BB, brachialis (BR), and brachioradialis (BRD) were considered for estimating the integrated force at the wrist. Considering the handgrip force as the prediction object, Hoozemans et al. compared the performance of a section of forearm muscle and the combinations of three, four, five, and six forearm muscles. They observed that all the combinations outperformed the application of an individual section of muscle (Hoozemans and van Dieen, 2005). Although certain progress has been achieved, the investigation of force estimation based on multiple muscles is relatively preliminary. It is necessary to investigate whether an individual muscle or a combination of muscles is appropriate for force estimation.

Various force estimation models have been developed in the literature. Specifically, the Hill Type model (Hill, 1938), polynomial model (Huang et al., 2017), fast orthogonal search (Mobasser et al., 2007; Chen X. et al., 2018), and simple artificial neural network (Luh et al., 1999; Bai and Chew, 2013; Wu et al., 2017) have been adopted successfully to establish the relation between EMG and the end-effector force. In recent years, deep learning algorithms have also been introduced to the field of force estimation. Xu et al. (2018) used convolutional neural network (CNN), long short-term memory (LSTM) network, and their combination (C-LSTM) to predict the end-effector force generated by static isometric elbow flexion. They achieved highly accurate, subject-independent force estimation (Xu et al., 2018). Choi et al. (2010) mapped sEMG signal to force by a deep artificial neural network and achieved good performance in real-time pinch force estimation. In above researches, only individual skeletal muscle was considered for force estimation. It is generally acknowledged that deep learning frameworks can extract features from raw data without handcrafted feature selection. In the deep architecture, the output of each layer, which contains all the information from the input data, can be considered as the deep fusion of the original data. For multi-muscle contraction task, a key problem is effectively fusing the activation information of different muscles for force estimation. Consequently, deep learning algorithms exhibit the potential for fusing the contraction characteristics of multiple muscles for realizing highly accurate force estimation.

To explore whether the individual muscle or the combination of muscles is more suitable for the end-effector force estimation during multi-muscle contraction tasks, this paper carried out a novel HD-sEMG-based force estimation research. The main features of this research are as follows: (1) To fuse the activation information from different muscles effectively, DBN was adopted to establish the relation between HD-sEMG and the generated force. (2) Based on the well-trained DBN force estimation model, an index was presented to evaluate the priority of muscles for estimating the generated force. (3) Taking elbow flexion task and palm-pressing task as examples, the priority of the BB, BR, TB, BRD, and extensor digitorum communis (EDC) for estimating the generated force were investigated.

## Materials and Methods

**FIGURE 1:**
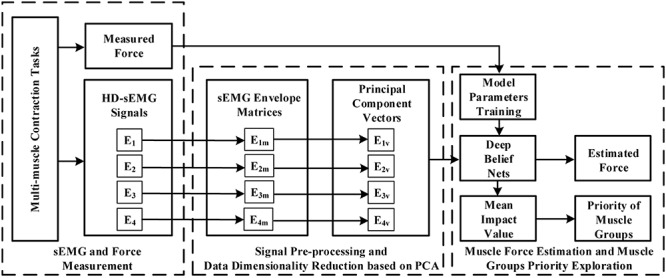
Block diagram of proposed force estimation framework.

The block diagram in Figure 1 demonstrated the overall research route of this study. When the multi-muscle contraction tasks were performed, HD-sEMG signals were collected from four primary areas of the upper arm and forearm. These areas mainly included muscles of the BB, BR, TB, BRD, and EDC. The representative signals of the four primary areas, which were considered as the input signal of the force estimation model, were extracted from HD-sEMG by principal component analysis (PCA) algorithm. They were then fed separately or together into the DBN to estimate the generated force. Finally, the priority of individual muscle groups for estimating the generated force was analyzed with an index termed the mean impact value (MIV).

### Two Multi-Muscle Isometric Contraction Tasks and Data Collection

In this study, 13 right-handed male participants aged 22–27 years (and without neural or musculoskeletal diseases) voluntarily participated. Nine subjects participated Task 1, but part of them were not involved in Task 2 due to personal time arrangement, and some new subjects were recruited for sufficient experimental data in Task 2. Overall, the amount of total subjects is 13 and the subjects in two tasks were not same entirely. All the participants were informed of the experimental procedures and signed an informed consent approved by the Ethics Review Committee of First Affiliated Hospital of Anhui Medical University (No. PJ 2014-08-04).

**FIGURE 2:**
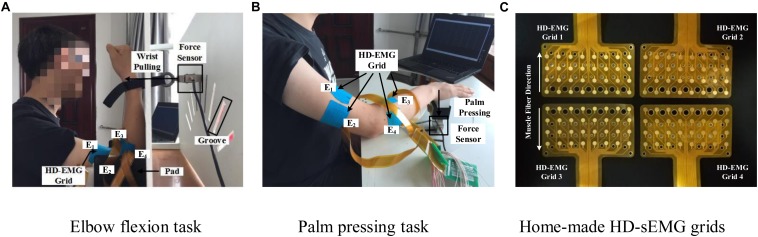
Two multi-muscle contraction tasks and home-made HD-sEMG grids. **(A)** Elbow flexion task **(B)** Palm pressing task, and **(C)** Home-made HD-sEMG grids.

The main feature of isometric contraction is that no contraction movement is occurred during the contraction and the length of muscle fiber is not changed. Because they are relatively simple to perform, isometric tasks are usually targeted as the research subject in related muscle force prediction researches. In this study, we also designed two specific isometric tasks as the research subjects. The two multi-muscle contraction tasks are described as follows: (1) *Elbow Flexion Task:* As shown in Figure 2A, during this task, the participants were seated upright on a chair. Their right forearm clung vertically to the front layer of an apparatus, whereas the elbow joint was placed at 90°. The wrist was connected tightly to the force sensor (LAS-B, Norson, China), which was fixed in the groove of the back layer of the apparatus. The participants were asked to perform elbow flexion with the wrist pulling force, following the guiding force displayed on the screen from a human–computer interaction interface. In total, 9 of the 13 participants conducted the elbow flexion task. (2) *Palm-Pressing Task*: As shown in Figure 2B, during this task also, the participants were seated upright on a chair. Their right forearm clung horizontally to the front layer of the apparatus, and the palm was placed closely above the force sensor. The participants were asked to perform a palm-pressing task with the press force following the guiding force. In total, 10 of the 13 participants conducted the palm-pressing task. Concretely, during the experiment, the participants were asked to perform the two contraction tasks by contracting BB, BR, TB, BRD, and EDC as much as possible. This setup guaranteed that other muscles (including shoulder muscles) were not basically involved in the contraction. In addition, based on our previous investigation on the muscle force estimation in dynamic random scenario (Hu et al., 2019), sinusoidal mode which have the potential to make the model generalized for any other force pattern, was selected as the target force mode in this study.

Custom-built apparatus (shown in Figure 2) was used to aid the participants in performing the two multi-muscle contraction tasks. Four pieces of in-house manufactured HD-EMG grids (E_1_, E_2_, E_3_, and E_4_; shown in Figure 2C) were used to collect HD-sEMG data. Each HD-EMG grid contains 32 electrodes (four rows and eight columns) with 3 mm diameter and 10 mm inter-electrode interval, covering a collection surface of 8 × 4.6 cm. The carrier material of the HD-EMG array is a polyimide flexible material, so that all the electrodes could fit well with the skin. Surface EMG signals were amplified by a factor of 1371.1, and then physically filtered using a 20–500 Hz band-pass (Huang et al., 2017). The skin of the front and back of both upper arm and forearm was wiped with alcohol to reduce skin–electrode impedance. As shown in Figure 2B, four arrays were placed to cover the muscles BB and BR (E_1_), TB (E_2_), BRD (E_3_), and EDC (E_4_) of the right arm. The mode of signal acquisition is monopolar, i.e., the signals are differences between the measured electrodes and reference electrode. The reference electrode was self-adhesive and attached to the back of the right hand. A ground electrode that could reduce the interference from a 50 Hz power line was attached to the back of the left hand. Both force and sEMG signals were sampled at 1 kHz using a 16 bit A/D converter (ADS1198) (Huang et al., 2017).

At the beginning of each data collection experiment, participants were first asked to perform maximal voluntary contraction (MVC) to produce the maximal wrist pulling force (elbow flexion task) or maximal palm-pressing force (palm-pressing task). Each participant repeated the MVC tasks three times, and the maximum one was selected as the MVC value. During the experiment, the participants were asked to perform a specific task in sinusoidal mode force using the right arm. Each task was carried out at three force levels with the amplitude of sinusoidal force ranging from 0–20%MVC, 0–40%MVC, and 0–60%MVC, respectively. The duration of the sinusoidal mode force was 6 s. The trail was repeated 10 times at each force level for each participant. The data used for training and testing of the model contained eight repetitions. The other two repetitions were adopted for validation. To aid the participants in better completing the contraction protocols in sinusoidal mode force, the real-time feedback of the force-tracking curve and the target force were displayed on a human–computer interaction interface. It is noteworthy that all the participants were asked to practice each muscle contraction protocol until they could perform the tasks according to the experimental requirements. All the data were saved to a disk for off-line analysis by Matlab R2016a.

### Signal Pre-processing and Representative Activation Signal Extraction Based on PCA Algorithm

Raw HD-sEMG signal was preprocessed to promote the signal quality according to the following procedures. First, some channels whose signal amplitude was below or beyond the reasonable range, or the random noise interference exist would be discarded and replaced by the mean value of neighboring channels. Then, the signals were high-pass filtered (finite impulse response filter, cutoff frequency 20 Hz, Hanning window, 80th order) to remove the low frequency noise. The envelope of each channel was obtained by full-wave rectification and moving average filtering (window size 100 ms) (Popovic et al., 2016). In each contraction cycle, the measured force signals were normalized using the maximum value.

For reducing the number of input units of the force estimation model and saving computation cost, the mean-removed envelope matrix of each HD-sEMG grid was decomposed by the PCA algorithm for dimensionality reduction in this study. That is, the PCA algorithm was used to extract the representative activation signals from each HD-sEMG grid. The processing by PCA is to transform a mean-removed sEMG envelope matrix *X* = [*X_1_, X_2_*,., X_*M*_] (*M* represents the number of channels) into a matrix *Y* = [*Y_1_,Y_2_*,., Y_*N*_] consisting of a series of uncorrelated principal components or modes by orthogonal transformation technique (Wold et al., 1987; Abdi and Williams, 2010). The first principal component *Y*_1_ accounting for the highest variance can be represented by the linear combination of *X_1_, X_2_*,., X_*m*_ as shown in Formula (1) (Wold et al., 1987; Abdi and Williams, 2010).

(1) $$Y_{1}=a_{11}\,X_{1}+a_{12}\,X_{2}+\cdots +a_{1\,M}\,X_{M}$$

The second principal component *Y*_2_, which accounts for the next highest variance, can be calculated similarly. The process continues until *M* principal components, whose amount equals to the number of channels, have been calculated. Consequently, the transformation of the original signal *X* to the principal component matrix *Y* can be described by Formula (2) (Wold et al., 1987; Abdi and Williams, 2010):

(2) Y=A⁢X

The rows of the matrix *A* are the eigenvectors of the covariance matrix *X*. The elements of each eigenvector are the weights. The corresponding eigenvalues are the variance explained by each principal component, which decreased monotonically from the first principal component to the final one (Wold et al., 1987; Abdi and Williams, 2010). The principal component with larger eigenvalues is accompanied by larger energy. Therefore, it also includes substantial valid information of the original data. In many studies, the principal components whose cumulative variance explained contribution rate attains 0.85 were generally selected to reflect the information and characteristics of the original data (Jeffers, 1967; Chen et al., 2000; Webster, 2001). In this study as well, the threshold of variance explained was set to 0.85. The principal components, which were extracted from each HD-sEMG array and satisfied the criteria, were selected as the representative activation signal of the corresponding array.

### sEMG-Force Relation Establishment Based on Deep Belief Network

#### Deep Belief Network

**FIGURE 3:**
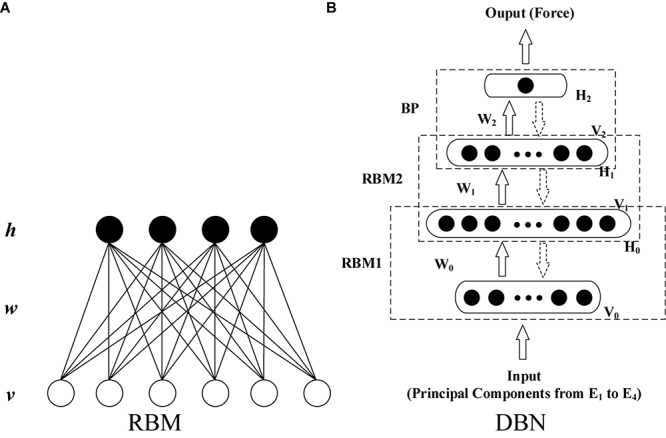
Typical topological structure of RBM and DBN. **(A)** RBM and **(B)** DBN.

Deep belief network (DBN) is a layer-by-layer network constructed by stacking multiple layers of restricted Boltzmann machines (RBMs) (Hinton, 2002; Hinton et al., 2006). It exhibits higher generative modeling capability than other shallow architectures even for a marginal amount of sample data. As shown in Figure 3A, RBM is a two-layer, undirected, and energy-based model. The visible units in the bottom layer represent observations and are connected to the hidden units, which represent the abstract features. The detailed formula derivation of RBM was presented in Supplementary Appendix I.

With the unsupervised learning algorithm for RBM, the training of a DBN can be implemented as two steps: layer-wise pre-training and fine-tuning. The first step is to train a stack of RBMs recursively and rapidly layer by layer. This produces a series of initial net parameters. After pre-training, the RBMs are unfolded to establish the DBN model with the initial parameters. Then, the gradient-based optimization algorithm is applied further to minimize differences between the input and output data (Hinton, 2002; Hinton et al., 2006; Xiong et al., 2015; Su et al., 2016; Chen J. C. et al., 2018).

In this study, a DBN that has four layers (including two hidden layers) was constructed (shown in Figure 3B) to establish the EMG–force relationship. The inputs of the DBN model were the principal components after min–max normalization extracted using the minimum and maximum absolute values among all the extracted principal components from four HD-sEMG arrays. Consequently, the number of input units was determined by the number of principal components. To ensure the consistency of the structure of neural network, for all the participants, the minimum amount of principal components that satisfied the threshold of variance explained was selected as the number of input units in the model training phase. In the testing phase, the threshold of variance explained was not considered. Moreover, the numbers of principal components and model input units were kept accordant with those in the training phase. The output layer contains one unit. The number of units in two hidden layers was optimized during the model establishment and parameter adjustment. Except for the training data and testing data, the validation data was set to prevent the model over fitting to the training data.

#### Evaluation Index of Significance of Individual Muscle Group to Integrated Force

In many studies, the MIVs of the artificial neural network were used to evaluate the importance of different inputs to the output of the model (Dombi et al., 1995). The significance of an individual muscle group for force estimation was explored in this study by evaluating the effect of different model inputs on the output. Specifically, based on a well-trained DBN model, each model input was marginally altered sequentially. In addition, the influence of different inputs was evaluated by comparing the variation in the model output. The procedures are provided in detail as follows.

Assume that the sample matrix *P_m__×__n_* = [*P*_1_, *P*_2_, …, *P*_*m*_]^T^ represents the model input signals of a contraction cycle. Here, *m* is the number of principal components extracted from all the four arrays, and *n* is the number of samples. The DBN was trained with *P*_*m*__×_*_*n*_* and the corresponding measured force signal. After the model was established, the principal component *P*_1_ was increased and decreased each by 10% × *P*_1_ to obtain two new training vectors *P′*_1_ and *P*^”^_1_. The other principal components (from *P*_2_ to *P*_*m*_) remained unaltered. The two new sample matrixes [*P′*_1_, *P*_2_, …, *P*_*m*_]^T^ and [*P*^”^_1_, *P*_2_, …, *P*_*m*_]^ T^ were input to the well-trained DBN model to obtain two results *R′*_1_ and *R*^”^_1_. Then, the mean difference of *R′*_1_ and *R*^”^_1_, which is defined as the MIV of *P*_1_, was calculated using Formula (3) (Dombi et al., 1995; Liu et al., 2012; Qi et al., 2016):

(3) $$M\,I\,V_{1}=\sum_{n}\left(R_{1}-R_{1}\right)/n$$

MIV_2_, …, MIV*_*m*_* was calculated similarly. All the principal components were sorted according to the absolute values of the MIVs to obtain the relative impact of each model input on the output. The one corresponding to the largest MIV exerted the most important influence on the output (Dombi et al., 1995; Liu et al., 2012; Qi et al., 2016). If over one input corresponds to a muscle group, the maximum MIV of these input units was considered as the final index for this muscle group. Consequently, the MIVs of model input signals extracted from each array can reflect the relative significance of individual muscle groups to the integrated force.

### Evaluation Parameters of Muscle Activation Level

In each contraction cycle, HD-sEMG signals were normalized using the maximum absolute value of all the 128 channels. Moreover, the root mean square (RMS) value of each normalized HD-sEMG channel was calculated first. Then, the sum of square of RMS in a contraction cycle was defined as the activation level according to Formula (4). *RMS*_*j*_ (*j* = 1, 2, 3, 4) represents the RMS of the *j*th array in a contraction cycle, *i* (*i* = 1, 2, …, 32) is the channel number, and *k* represents the force level (20, 40, and 60%MVC).

(4) $$I_{j}^{k}=\sum_{i=1}^{32}R\,M\,S_{j}^{k}\,{\left(i\right)}^{2}$$

### Statistical Analysis

The root mean square difference (RMSD) (Huang et al., 2017) and goodness of fit (*R*^2^) (Zhang et al., 2018) between the normalized measured force and estimated force were selected to evaluate the performance of the proposed end-effector force estimation framework. In Formulas (5) and (6), y and ỹ are the normalized measured force and estimated force, respectively. *N* is the number of samples, and $\bar{y}$ is the mean of the measured force of the *N* samples.

The analysis of variance (ANOVA; SPSS 22, Chicago, IL, United States) was used for statistically analyzing the experimental results. The fixed factor “HD-sEMG grid” was tested. The dependent variables were RMSD, *R*^2^, and MIV. The null-hypothesis of the ANOVA test for RMSD and *R*^2^ was that there is no difference in the force estimation accuracy among different grids. The null-hypothesis for MIV was that there is no difference in the impact on end-effector force among different grids. The significance level was 5%.

(5) $$R\,M\,S\,D=\sqrt{\frac{\sum_{i=1}^{N}{\left[y\,\left(i\right)-\tilde{y}\,\left(i\right)\right]}^{2}}{N}}$$

(6) $$R^{2}=1-\frac{\sum_{i=1}^{N}{\left[y\,\left(i\right)-\tilde{y}\,\left(i\right)\right]}^{2}}{\sum_{i=2}^{N}{\left[y\,\left(i\right)-\bar{y}\right]}^{2}}$$

## Experimental Results and Analysis

### Muscle Group Activation State Analysis

**FIGURE 4:**
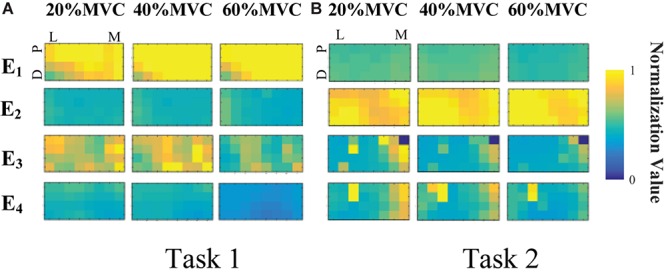
RMS maps of four HD-sEMG grids in typical contraction cycles from Participant 1 (D, Distant; P, Proximal; L, Lateral; M, Medial). **(A)** Task 1. **(B)** Task 2.

**FIGURE 5:**
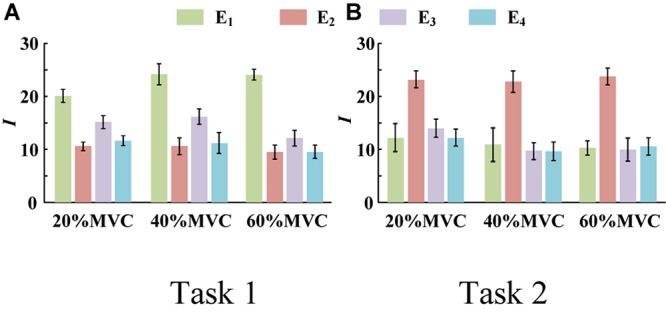
Activation level *I* of four muscle groups of all participants. (9 participants for Task 1 and 10 participants for Task 2. Error bars represent the standard deviation.) **(A)** Task 1 and **(B)** Task 2.

Figure 4 demonstrates the RMS maps of the four HD-sEMG grids in typical contraction cycles of two tasks at three target force levels, for a representative participant (Participant 1). In the two sub-figures, each row corresponds to an array, and each column corresponds to force level. The electrode arrangement is consistent with the actual HD-sEMG grid. It should be indicated that the normalization was implemented among the four HD-sEMG grids in each contraction cycle. Therefore, the RMS maps can reflect only the relative differences in muscle activation at each force level. Figure 4A reveals that the activation intensities of the BB and BR seem to be higher than those of the BRD, TB, and EDC at all the three force levels in Task 1. From the discrepancy of the activation intensity of each muscle group, the BB and BR are always in the dominant activation state at the three force levels. Moreover, the BRD is in the co-activation state at 20%MVC and 40%MVC. In Task 2, as shown in Figure 4B, the activation intensity of the TB is higher than that of the other muscles. Furthermore, certain scattered areas of the BRD and EDC are activated at the middle and low force levels. The activation level results of the four HD-sEMG grids of all the participants are illustrated in Figure 5. In Task1, for all the participants, the activation level of the BB and BR is higher than that of the other muscle groups. In Task 2, the TB is always in the state of high activation level. The experimental results reveal that the muscle synergy or muscle co-activation situation generally appears at the middle and low force levels and that the impact of the dominant muscle on the target task is remarkable at a high force level.

### Representative Activation Signal Extraction Results

When the PCA algorithm was applied to the normalized and mean-removed envelope matrix of each HD-sEMG grid, it was observed that for almost all the data at the three force levels of all the participants, the first principal component can attain the threshold standard of variance explained (0.85). Consequently, only the first principal components of each array were selected as the representative signals. These were then normalized by the minimum and maximum values among the four principal components as the input signals of the force estimation model.

**FIGURE 6:**
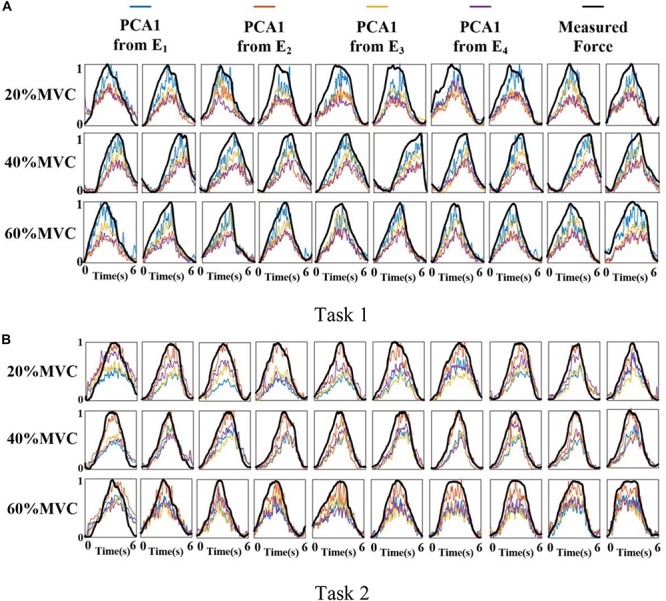
Normalized activation signals extracted from four arrays and measured force of Participant 1 (10 typical contraction cycles are provided at each force level). **(A)** Task 1 and **(B)** Task 2.

**FIGURE 7:**
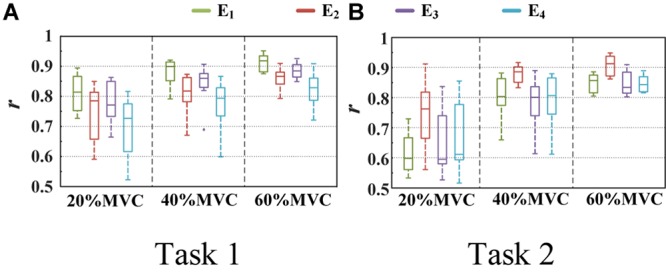
Correlation coefficient between different activation signals and measured force. (9 participants for Task 1 and 10 participants for Task 2.) **(A)** Task 1 and **(B)** Task 2.

Figure 6 shows the representative activation signals extracted from the four primary muscle groups and the corresponding measured force for Participant 1 in two tasks. The results of 10 typical contraction cycles at each force level (20, 40, and 60%MVC) are provided. Table 1 demonstrates the correlation coefficient (*r*) between the extracted activation signals and measured force. Figure 7 shows the results of the correlation coefficient (*r*) between the extracted activation signals and measured force for all the participants. For both the tasks and four muscle groups, *r* increases gradually when the force level increases. In Task 1, the correlation between the activation signal extracted from the BB and BR and the measured force is higher than that of the other muscle groups. In Task 2, the activation signal extracted from the TB is more related to the measured force compared with the other three muscle groups. Combining the results of the correlation analysis and the muscle activation state analysis, it is concluded that the muscles that are activated strongly in the task are more related to the generated force.

**TABLE 1:** Correlation coefficients between extracted activation signals and measured force for Participant 1.

***r* (mean ± std)**	**Task 1**			**Task 2**		
	**20%MVC**	**40%MVC**	**60%MVC**	**20%MVC**	**40%MVC**	**60%MVC**
E_1_	0.82 ± 0.03	0.90 ± 0.02	0.94 ± 0.01	0.61 ± 0.01	0.76 ± 0.03	0.85 ± 0.02
E_2_	0.75 ± 0.02	0.81 ± 0.02	0.88 ± 0.02	0.74 ± 0.02	0.84 ± 0.02	0.92 ± 0.02
E_3_	0.79 ± 0.02	0.86 ± 0.01	0.90 ± 0.02	0.64 ± 0.01	0.77 ± 0.02	0.86 ± 0.02
E_4_	0.72 ± 0.03	0.77 ± 0.02	0.82 ± 0.07	0.62 ± 0.01	0.75 ± 0.01	0.83 ± 0.03

### Force Estimation Results

For the two multi-muscle contraction tasks (elbow flexion task and palm-pressing task), two types of force estimation experiments were carried out with the leave-one-out cross validation method for verifying the feasibility of the proposed force estimation framework, and the statistical analysis was also based on the results of force estimation with cross validation. First, HD-sEMG signals from all the four muscle groups and an individual muscle group were used to estimate the respective integrated force. Then, based on the DBN model well-trained with signals from all the four muscle groups, the MIVs of the four muscles were calculated, and the impact of the different muscle groups on the target force was investigated. The number of hidden layer units of DBN was adjusted according to the training error and generalization error. When the training error was convergent and generalization error decreased to a relatively lower level, the number of hidden layer units was the optimal selection. We have tried one, two, and three hidden layers, and found that double hidden layers can well meet the needs of high recognition rate and low computation cost. For the number of neural units in each layer, we have tried 32–128 units, and found that 80 units for one-dimensional input and 100 units for four-dimensional input were the appropriate selection, which was easy to converge and has a higher recognition rate meanwhile. Finally, when individual muscle group was input, four layers of DBN were set to 1, 80, 80, 1 respectively; when four muscle groups were input, four layers were set to 4, 100, 100, 1 respectively. All following results were based on the test phase of the DBN analysis.

**FIGURE 8:**
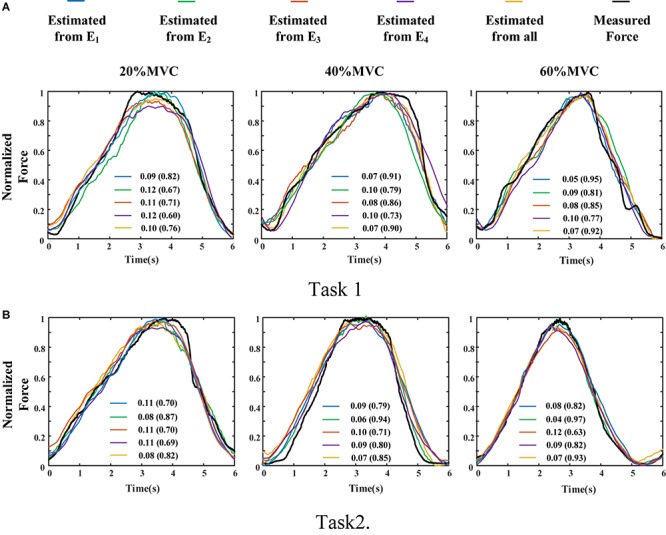
Illustration of force estimation results for Participant 1 in a contraction cycle. The statistical values shown in each sub-graph are presented in the form of RMSD (R^2^). **(A)** Task 1 and **(B)** Task 2.

The measured force and estimated force in a contraction cycle (6 s trail) of Participant 1 are shown in Figure 8 as an example. It should be note that Participant 1 did not always perform best among all subjects, but in the top quarter among all participants. It is evident that using the signals from an individual muscle and from all the four muscles obtained different force estimation performances. In Task 1, the force estimation performance of E_1_ (BB and BR) was the highest (RMSD = 0.0918 for 20%MVC, 0.0765 for 40%MVC, and 0.0593 for 60%MVC) and that of the combination of all the four muscle was the second (RMSD = 0.1040 for 20%MVC, 0.0794 for 40%MVC, and 0.0740 for 60%MVC). In Task 2, the best estimation was from E_2_ (TB) (RMSD = 0.0836 for 20%MVC, 0.0699 for 40%MVC, and 0.0429 for 60%MVC), and the suboptimal estimation originates from the combination of the four muscles (0.0894 for 20%MVC, 0.0740 for 40%MVC, and 0.0731 for 60%MVC). In particular, at the high contraction force level of 60%MVC, the estimation performances using signals from the BB and BR or TB are superior.

**FIGURE 9:**
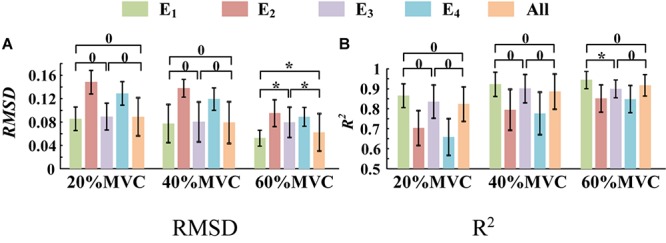
Force estimation results for Task 1. (9 participants, ^0^*p* ≥ 0.05, *p < 0.05, the statistical analysis results are marked only among “E_1_,” “E_3_,” and “All” for higher visibility. Error bars represent the standard deviation.) **(A)** RMSD and **(B)** *R*^2^.

**FIGURE 10:**
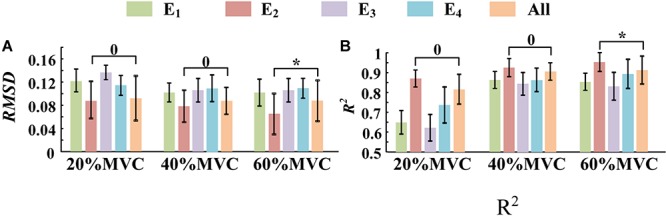
The force estimation results for Task 2. (10 participants, ^0^*p* ≥ 0.05, *p < 0.05, the statistical analysis results are only marked between “E_2_” and “All” for better visibility. Error bars represent the standard deviation.) **(A)** RMSD and **(B)** R^2^.

A one-way ANOVA was performed for two tasks and the result was shown in Table 2. Across two tasks, a significant RMSD difference (*p* < 0.05) and *R*^2^ difference (*p* < 0.05) both occurred among different grids. As the fixed factor was significant, *post hoc* multiple comparisons were executed. The force estimation results and the pairwise *post hoc* tests of the two tasks for all the participants were presented in Figures 9, 10, respectively. With regard to Task 1, E_2_ and E_4_ exhibit low performance. Therefore, the statistical analysis results are marked among only “E_1_,” “E_3_,” and “All” for higher visibility. “All” represents all representative signals from E_1_ to E_4_ were fed into DBN together as the four-dimensional input. At 20 and 40%MVC, the force estimation performances (both RMSD and R^2^) do not exhibit significant discrepancy between E_1_, E_3_, and all arrays. However, E_1_ is significantly higher than the other scenarios at 60%MVC in the results of 0.0392–0.0641 RMSD and 0.8809–0.9817 *R*^2^ (*p* < 0.05). For Task 2, the performances of E_1_, E_3_, and E_4_ are inferior. Therefore, the statistical analysis results are marked only between “E_2_” and “All” for higher visibility. At 20 and 40%MVC, good force estimation performance was obtained for both E_2_ and all arrays. However, E_2_ is significantly higher than all arrays at 60%MVC (*p* < 0.05). At 60%MVC, the results of 0.0189–0.0925 RMSD and 0.9112–0.9918 *R*^2^ obtained from E_2_ were the best among those of the all the scenarios. Based on the above results, we can conclude that the highest force estimation performance was not always obtained by considering the HD-sEMG signals from all the four muscles involved. Dominant muscles can better describe the generated force characteristic in multi-muscle related contraction task.

**TABLE 2:** Results of the one-way ANOVA on RMSD, R^2^ with different HD-sEMG grids as fixed factors.

**Task (with different force levels)**		**Significance (*p*)**	
		**RMSD**	***R*^2^**
1	20%MVC	0.000139*	0.000452*
	40%MVC	0.000354*	0.007224*
	60%MVC	0.004166*	0.011007*
2	20%MVC	0.004003*	0.000006*
	40%MVC	0.003761*	0.007249*
	60%MVC	0.002229*	0.009139*

*9 participants for Task 1 and 10 participants for Task 2, ^0^p ≥ 0.05, ^∗^p < 0.05.*

**FIGURE 11:**
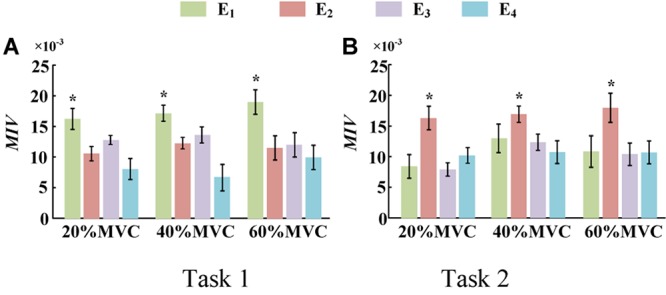
MIVs for all participants. (9 participants for Task 1 and 10 participants for Task 2, *p < 0.05, the statistical analysis mark represents that the value of this factor is significantly higher than all the others. Error bars represent the standard deviation.) **(A)** Task 1 and **(B)** Task 2.

To demonstrate the effects of the different muscles on the force estimation further, the MIVs from Participant 1, which were calculated based on the well-trained DBN, are presented in Table 3. The contraction cycles are in accordant with those in Figure 8. MIV_1_, MIV_2_, MIV_3_, and MIV_4_ correspond to E_1_, E_2_, E_3_, and E_4_, respectively. The MIVs across all the participants are shown in Figure 11. The one-way ANOVA was also implemented. Combining the results of force estimation using an individual muscle, it is observed that the MIV can reflect the influence of individual muscle groups on the generated force. In Task 1, the MIVs of the input units corresponding to E_1_ and E_3_ are higher than those of the other two arrays at the low and middle force levels. E_1_ has the largest value (*p* < 0.05) at all the three force levels. Therefore, the BB and BR can be considered to exert the largest effect on the generated force. In Task 2, the MIV of the input unit corresponding to E_2_ has the largest value (*p* < 0.05) at all the three force levels. Therefore, the TB is always the main contributing muscle, and the other three muscle groups exert less impact on the generated force compared to the TB. In summary, according to the ranking of the MIVs, in multi-muscle contraction tasks, the priority of different muscle groups suitable for force estimation can be obtained.

**TABLE 3:** MIV results in a typical contraction cycle of Participant 1.

**MIV**	**Task 1**			**Task 2**		
	**20%MVC**	**40%MVC**	**60%MVC**	**20%MVC**	**40%MVC**	**60%MVC**
M	0.0	0.01	0.01	0.00	0.0132	0.0107
IV_1_	168	71	74	86		
M	0.0	0.01	0.01	0.01	0.0170	0.0182
IV_2_	122	32	13	64		
M	0.0	0.01	0.01	0.00	0.0134	0.0100
IV_3_	144	46	20	79		
M	0.0	0.00	0.00	0.01	0.0101	0.0103
IV_4_	085	76	94	09		

## Discussion

In this study, a research on the end-effector force estimation in multi-muscle contraction task was carried out. In particular, two types of multi-muscle contraction tasks were considered as the research objects. In addition, whether a combination of muscles or an individual muscle is more suitable for the end-effector force estimation in a multi-muscle contraction task was explored. The main contribution, limitations, and future work are summarized and discussed as follows.

### Performance of the Deep Learning Algorithm-Based Force Estimation Framework

To achieve highly accurate force estimation in multi-muscle contraction tasks, a framework based on HD-sEMG and DBN was proposed in this study. To our knowledge, although DBN is one of the mainstream architectures widely used in the artificial intelligence field, there are few researchers using it to undertake force estimation. The results of force estimation experiments using an individual muscle and those using multiple muscles verify the feasibility and effectiveness of the proposed framework. The minimum RMSD of 0.0189 (corresponding *R*^2^ of 0.9804) was achieved for the elbow-flexion force estimation. An average RMSD of 0.0724 was obtained among all the repetitions in the two tasks when using only the co-activated or dominant muscle. In a work of Staudenmann et al. (2006), the end-effector force was estimated by the sEMG of TB during the isometric elbow extension. PCA algorithm was used to discard redundant information and noise in the HD-sEMG signals, and a minimal RMSD of 0.0940 was obtained. In a work using sEMG obtained from several forearm muscles to estimate the palmar pinch force by an artificial neural network, the force estimation error was 0.081 ± 0.023 RMSD (Choi et al., 2010). Considering these results, the performance of the proposed force estimation framework is effective.

However, the proposed framework does not achieve the highest performance reported in the available literature. For example, in the work of Harrach et al. (Al Harrach et al., 2017), three elbow flexors including the BB, BR, and BRD were jointly considered to estimate the generated force at the wrist. A remarkably low RMSD (approximately 0.0121) was obtained at 90%MVC for a participant. Related research (Gandevia and Kilbreath, 1990; Staudenmann et al., 2006; Al Harrach et al., 2017; Huang et al., 2017) has demonstrated that because the relative signal-to-noise ratio increases with the muscle activation level, the higher is the muscle contraction level, the higher is the force estimation accuracy. In this study, the largest target force level is 60%MVC. We consider that the relatively low estimation performance may be related to the low muscle contraction level.

For force estimation in multi-muscle contraction tasks, the fusion of the activation information from multiple muscles and the establishment of the non-linear relationship between sEMG and the end-effector force are two critical issues. The frequently used force estimation model in the past decades consisted mainly of physiological models (e.g., Hill Type model; Hill, 1938) and mathematical models (e.g., polynomial model; Huang et al., 2017) and fast orthogonal search (Mobasser et al., 2007; Chen X. et al., 2018). Although these traditional models can address the simple regression fitting for force prediction under certain requirements of precision, they more or less exhibit limitations. The polynomial model and fast orthogonal search are both incapable of fusing multiple homologous sEMG signals effectively. With regard to the Hill type model, the physiological parameters need to be measured by ultrasound or other means for each participant. Moreover, it is challenging to determine the relationship among the muscle force, muscle length, and contraction velocity over time during a complex contraction task. Neural networks based on deep learning algorithm can theoretically approximate any non-linear function to fit the relationship between force and EMG to the extent feasible. That is, they have the potential to capture the overall outline and local details of the force profile for highly accurate force estimation. However, deep learning algorithm also has some limitations. For example, the demand of sample size for model training is large, the calculation and time cost of network training is high, and the interpretability of the hidden layer is poor.

Although two specific isometric tasks was targeted to verify the performance of the proposed framework. The DBN-based force estimation method is not only designed for the isometric contraction task, but also can be extended to other types of muscle contraction tasks. Because the neural network is effective for data fitting and has the good generalization ability, so as long as the contraction task does not change much (such as just changing the profile of the force, or adding some simple dynamic scenarios), only fine-tuning the parameters of the network using diverse training data is needed, rather than changing the structure. When the amount of muscles involved in the contraction task is changing, different model inputs would lead to different amount of input units and hidden layer units. In generally, up to five hidden layers would be tried, the one that can well meet the needs of high recognition rate and low computation cost would be considered as the final selection. The number of hidden layer units of DBN can be adjusted according to the training error and generalization error. When the training error is convergent and generalization error decreases to a relatively lower level, the number of hidden layer units is the optimal selection.

In addition, only sinusoidal shaped force profile was targeted in this study. Because no *a priori* information about the force profile was considered in the design of the network, the prediction of the model was only determined by the quality of training process. The reason for the selection of sinusoidal shaped force profile is that it has a good coverage for different force patterns. As shown in Supplementary Appendix II, taking the Task 1 as an example, force estimation experiments were supplemented on two new contraction tasks with force profiles termed ramp and hold (R&H) pattern and staircase (or piecewise constant) pattern respectively. The experimental results confirmed that the model trained with data of sinusoidal force pattern can be used for force estimation in a new force pattern that never occurred in the training data.

Finally, it is important to point out that the usage of HD-sEMG is beneficial for improving force estimation performance. In general, the attachment of the HD-sEMG grid does not need to be as precise as the discrete electrode, so a slight deviation will not affect the experimental results. However, we still checked the approximate position of each muscle via the knowledge of human anatomy to ensure that the target muscles could be covered as much as possible. In addition, the PCA algorithm was used to extract the main activation information of each muscle group, which will also reduce the impact of different electrode attachment positions.

### Preference for Force Estimation in Multi-Muscle Contraction Tasks: Individual Muscle or Multiple Muscles?

For multi-muscle contraction tasks, the generated force is the result of the contraction of multiple muscles. In theory, the highest performance should be obtained when the force estimation is carried out using activation information from all the involved muscles. However, contradictory research results exist with regard to whether an individual muscle or a combination of muscles is more suitable for force estimation. Hoozemans and van Dieen (2005) carried out a study on handgrip force estimation using different forearm muscles and their combinations. They observed that the performance of force estimation using the combinations were better than that obtained using any individual muscle. Chen X. et al. (2018) estimated the elbow force during static isometric elbow flexion using the HD-sEMG signals collected from the upper arm muscles. Their experimental results demonstrated that compared with the combined use of agonist and antagonist, consideration of either the agonist or antagonist can improve the end-effector force estimation performance at different force levels (Chen X. et al., 2018). In the study of Gandevia and Kilbreath, they asked subjects to lift an object of standard weight; estimated the pulling force using the hand muscle (first dorsal interosseous), forearm muscle (flexor pollicis longus), upper limb muscle (elbow flexor), and a combination of them; and observed that the upper limb muscle (elbow flexor) is more suitable for force estimation (Gandevia and Kilbreath, 1990).

In this study, two representative multi-muscle tasks were used as experimental research trials, and the problem of whether individual muscle or multiple muscles are more suitable for force estimation was investigated. First, the muscle activation analysis shows that the BB and BR (E_1_) are always in the dominant activation state at the three force levels in Task 1. The BRD (E_3_) is in the co-activation state at 20 and 40%MVC. The TB (E_2_) is always in the dominant activation state at the three force levels in Task 2. Second, the correlation coefficient analysis shows that the activation signal extracted from the key muscle can well track the variations of the measured force. In Task 1 (Figure 7A), the correlation between the activation signal extracted from the BB and BR and the measured force is higher than that of other muscle groups. In Task 2 (Figure 7B), the activation signal extracted from the TB is more related to the measured force compared with other three muscle groups. Third, the force estimation results show that the estimation accuracy obtained using the key muscle is similar or even better than that obtained using all muscles. In other words, the best force estimation performance could not always be obtained by taking the HD-sEMG signals from all the involved muscles. In Task 1 (Figure 9), the force estimation performance of E_1_ (BB and BR) was the highest and that of the combination of all the four muscle was the second. BRD (E_3_) outperformed the combination of four muscle groups when it was in the co-activation state at 20 and 40%MVC. In Task 2 (Figure 10), the best estimation was from E_2_ (TB), and the suboptimal estimation originated from the combination of the four muscles. Based on above results, we conclude that the dominant muscles with the highest activation level are more suitable for highly accurate force estimation than the combination of muscles.

The MIV index is proposed to evaluate the effect of an individual muscle group on the end-effector force. It was verified that it is feasible to rank muscle priority for force estimation in multi-muscle contraction tasks. The concept of measuring the contribution of different muscles to the generated force with the MIV index is innovative, and has practical application value in the field of biomechanics.

We try to interpret the experimental results from the perspective of physiology. First, according to the principles of physiology, BB and TB are an agonist-antagonist pair and play a significant role in the process of elbow flexion (Nordin and Frankel, 2001). Both experimental tasks of this study were in the state of elbow flexion. Because there was a trend of concentric contraction in Task 1, BB and BR were the main agonist muscles. For Task 2, there was a trend of eccentric contraction, so TB was the main agonist muscle (Jaskolski et al., 2007). The results of muscle activation level analysis was consistent with physiological view. Second, it was pointed out that, in some literature, although multiple muscles are involved in one contraction task, some of them only play the role of assisting and maintaining the force, and some contribute to the subtle fluctuations of the force (Gandevia and Kilbreath, 1990; Oliver et al., 2010). Combining Figures 5, 7, it is observed that the muscle activation signals with high activation level exhibited high correlation with the force curve. Thus, they could track the variation in the force more effectively. This result supports the physiological view as well.

In addition, we attempt to explain the phenomenon that high force estimation performance could be obtained by using the sEMG signals from the highly activated muscle from the perspective of neural network. According to Figure 11, we could observe that when all the muscle activation signals were considered, inputs with higher force correlation exerted higher impact on the output of the DBN. This phenomena revealed that the DBN network could concentrate its attention on the inputs with high force correlation.

### Limitations and Future Work

The main shortcoming of this study is that only two simple multi-muscle contraction tasks were investigated. The conclusions that dominant muscles can track variations in the generated force more effectively, and that the MIV index could be used to rank the muscles suitable for force estimation, need to be validated in more complex multi-muscle contraction tasks. Moreover, the ideal experimental condition in this study is uneasy to guarantee. Specifically, the shoulder muscles may participate in the contraction task more or less during the experiment. Furthermore, the utilization of a deep learning algorithm is still highly preliminary, and certain neural nets with an optimal net structure would be explored in the future for implementation of real-time and highly accurate force estimation. The sequential neural network, which can link the activation information at front and rear time-points, can be adopted to improve the force estimation accuracy in certain regular or periodic contraction tasks.

## Conclusion

To investigate whether an individual muscle or a combination of muscles is more suitable for the end-effector force estimation, a multi-muscle contraction force estimation framework is proposed. It was implemented on elbow flexion and palm-pressing tasks in this study. In the proposed framework, the relation between HD-sEMG and elbow flexion force/palm-pressing force was established using DBN. HD-sEMG was collected from four primary areas of the upper arm and forearm, mainly including muscles of the biceps brachii, brachialis, triceps brachii, brachioradialis, and EDC. The experimental results demonstrated that the dominant muscles with the highest activation degree could better track the variation in the generated force in a multi-muscle contraction task, and were more suitable for highly accurate force estimation than the combination of muscles. In addition, the proposed MIV index was effective for ranking muscle priority for force estimation in multi-muscle contraction tasks.

## Data Availability Statement

The datasets generated for this study are available on request to the corresponding author.

## Ethics Statement

The studies involving human participants were reviewed and approved by Ethics Review Committee of First Affiliated Hospital of Anhui Medical University (No. PJ 2014-08-04). The patients/participants provided their written informed consent to participate in this study.

## Author Contributions

RH designed the framework, completed the data collection and analysis, and wrote the manuscript. XC directed the research and substantially revised the manuscript. SC participated in the data collection and analysis. XZ and XC participated in the interpretation of the research results and manuscript revision. All the authors approved the final version of the manuscript.

## Conflict of Interest

The authors declare that the research was conducted in the absence of any commercial or financial relationships that could be construed as a potential conflict of interest.
