# Supplementary material for: Upper Limb End-Effector Force Estimation During Multi-Muscle Isometric Contraction Tasks Using HD-sEMG and Deep Belief Network
Source: Front Neurosci. 2020 May 7;14:450. doi: 10.3389/fnins.2020.00450 (PMC7221063; doi:10.3389/fnins.2020.00450)
Supplement: Supplementary file 1 [file Data_Sheet_1.docx]

**Appendix I：The Formula Derivation of RBM**

In the RBM model, the energy of the joint configuration can be calculated according to Formula (7) (Hinton 2002; Hinton et al. 2006):

$E\left( v,h \right)=-v^{T}wh-a^{T}v-b^{T}h=-\sum_{i=1}^{n_{v}} \sum_{j=1}^{n_{h}} w_{ij}v_{i}h_{j}-\sum_{i=1}^{n_{v}} a_{i}v_{i}-\sum_{j=1}^{n_{h}} b_{j}h_{j}$ (7)

where $w_{ij}$ is the interaction weight parameter between the visible unit $i$ and hidden unit $j$; $v_{i}$ and $h_{j}$ are the binary states of the corresponding units; $a_{i}$ and $b_{j}$ are the bias of the corresponding units; and $n_{v}$ and $n_{h}$ are the numbers of visible and hidden layer units, respectively.

The joint distribution of a binary RBM model is defined in Formula (8) (Hinton 2002; Hinton et al. 2006).

$p(v,h)=\frac{e^{-E(v,h)}}{\sum_{v,h} e^{-E(v,h)}}$ (8)

Owing to the absence of interaction terms between the visible and hidden units, the conditional probabilities $p(v|h)$ and $p(h|v)$ can be obtained using Formulas (9) and (10) (Hinton 2002; Hinton et al. 2006):

$p(h_{j}=1|v)= f(b_{j}+\sum_{i=1}^{n_{v}} w_{ij}v_{i})$ (9)

$p(v_{i}=1|h)= f(a_{i}+\sum_{j=1}^{n_{h}} w_{ij}h_{j})$ (10)

where $f(\cdot)$ is the activation function, which can be the *tanh* function.

By discretely summing up the hidden vectors, the marginal probability that the network assigns to the input samples can be described using Formula (11) (Hinton 2002; Hinton et al. 2006):

$p(v)=\frac{\sum_{h} e^{-E(v,h)}}{\sum_{v,h} e^{-E(v,h)}}$ (11)

The training process of RBM is to regard each training sample as a state vector and ensure that the probability of its occurrence is maximized, by adjusting the weights and bias. The derivatives of the logarithm marginal probability of the input data with respect to the weight coefficient and bias in the visible and hidden units are listed in Formulas (12), (13), and (14) (Hinton 2002; Hinton et al. 2006; Chen et al. 2018; Su et al. 2016; Xiong et al. 2015):

$\frac{\partial\log p(v)}{\partial w_{ij}}={<v_{i}h_{j}>}^{0} - {<v_{i}h_{j}>}^{k}$ (12)

$\frac{\partial\log p(v)}{\partial a_{i}}={<v_{i}>}^{0} - {<v_{i}>}^{k}$ (13)

$\frac{\partial\log p(v)}{\partial b_{j}}={<h_{j}>}^{0} - {<h_{j}>}^{k}$ (14)

where ${<v_{i}h_{j}>}^{0}$ represents the initial state of the visible and hidden units, and ${<v_{i}h_{j}>}^{k}$ is the equilibrium state*.* ${<v_{i}h_{j}>}^{k}$ can be calculated from the initial state by alternating Gibbs sampling and Markov chain for *k* iterations until the equilibrium.

The process includes substantial iterations of alternating Gibbs sampling, which requires k to be infinity to attain the equilibrium. However, Hinton proposed an efficient contrastive divergence (CD) algorithm with k = 1 to circumvent a significant amount of the computation required to obtain samples from the equilibrium distribution and to significantly reduce the variance that masks the gradient signal (Hinton 2002; Hinton et al. 2006). Therefore, the new learning rule attain the form of Formulas (15), (16), and (17) (Hinton 2002; Hinton et al. 2006; Chen et al. 2018; Su et al. 2016; Xiong et al. 2015):

$\Delta w_{ij}=\varepsilon({<v_{i}h_{j}>}^{0} - {<v_{i}h_{j}>}^{1})$ (15)

$\Delta a_{i}=\varepsilon({<v_{i}>}^{0}-{<v_{i}>}^{1})$ (16)

$\Delta b_{j}=\varepsilon({<h_{j}>}^{0} - {<h_{j}>}^{1})$ (17)

where $\varepsilon$ is the learning rate of the model.

# Appendix II：The Investigation on Models’ Generalization

Table I. The testing data and the training data.

| **Testing Data** | | **Training Data** |
| --- | --- | --- |
| Ramp and hold force pattern | 0-20%MVC | 0-20%MVC sinusoidal force pattern |
|  | 0-40%MVC | 0-40%MVC sinusoidal force pattern |
|  | 0-60%MVC | 0-60%MVC sinusoidal force pattern |
| Staircase force pattern | | 0-60%MVC sinusoidal force pattern |

(a) Ramp and hold pattern (b) Staircase pattern

Figure I. Two new contraction force patterns.

Taking the Task 1 as an example, force estimation experiments were supplemented on two new contraction tasks with force profiles termed ramp and hold pattern (Fig. I(a)) and staircase pattern (Fig. I(b)) respectively. In the ramp and hold pattern, the force level was increased linearly from 0% to a specific level in 3 seconds, and then held for 3 seconds. The task was carried out at three force levels with the amplitude ranging from 0–20%MVC, 0–40%MVC, and 0–60%MVC, respectively. In the staircase pattern, the force level was increased from 0% to 20%, 40%, and 60% MVC successively, and each targeted force level was held for 2 seconds. The duration of two pattern forces was both 6 s. The trail was repeated 10 times at each force level for all participants involved in Task 1 before. The model structure was adopted exactly the same as that in the main paper. The sinusoidal pattern force was used as training data as shown in Table I. The statistical results of muscle force prediction for all participants are shown in Fig. II and Fig. III. The measured force and estimated force in a contraction cycle (6 s trail) of Participant 1 are shown in Fig. IV as an example. Based on these results, we validated the generalizability of the proposed force estimation model for contraction task with different force profiles. And we believed that the model should be available for other complex force patterns in similar tasks including Task 2.

(a) RMSD (b) R^2^

**Figure II.** Force estimation results of ramp and hold force pattern. (9 participants, error bars represent the standard deviation.)

**Figure IV.** Illustration of force estimation results of two new experiments for Participant 1 in a contraction cycle. The statistical values shown in each sub-graph are presented in the form of RMSD (R^2^).

(a) RMSD (b) R^2^

**Figure III.** Force estimation results of staircase force pattern. (9 participants, error bars represent the standard deviation.)
